# Automatic Detection of Sinus Membrane Thickness Using a Hybrid Deep Learning Model Integrating Multiple Attention Mechanisms

**DOI:** 10.3390/jcm15186988

**Published:** 2026-09-09

**Authors:** Furkan Talo, Nurullah Duger, Burak Dagtekin, Mucahit Karaduman, Muhammed Yildirim, Tuba Talo Yildirim

**Affiliations:** 1 Department of Computer Engineering, Faculty of Engineering, Firat University, Elazig 23119, Turkey; 2 Department of Periodontology, Faculty of Dentistry, Firat University, Elazig 23119, Turkey; nduger@firat.edu.tr (N.D.); bdagtekin@firat.edu.tr (B.D.); taloyildirim@firat.edu.tr (T.T.Y.); 3 Department of Software Engineering, Malatya Turgut Ozal University, Malatya 44200, Turkey; mucahit.karaduman@ozal.edu.tr; 4 Department of Artificial Intelligence and Data Engineering, Firat University, Elazig 23119, Turkey; muhammedyildirim@firat.edu.tr

**Keywords:** artificial intelligence, attention, CNN, sinus membrane thickness, ViT

## Abstract

**Background/Objectives**: In dental implant surgeries, automatic detection and classification of maxillary sinus membrane thickening from Cone-Beam Computed Tomography (CBCT) images used for diagnosis and treatment planning, using an artificial intelligence-based system, will both reduce specialists’ error rates and enable faster processing. Therefore, in this study, a unique model was developed to automatically detect maxillary sinus membrane thickening from CBCT images. **Methods**: The first step of the developed model is data preprocessing. Then, feature extraction is performed using the backbone Convolutional Neural Network (CNN). The obtained features are processed in parallel with Squeeze-and-Excitation (SE), Convolutional Block Attention Module (CBAM), and Efficient Channel Attention (ECA) attention mechanisms. In this stage, channel, spatial, and local relationships are better represented. The combined features are passed to a Multilayer Perceptron (MLP) with dropout to mitigate overlearning, and the final class estimation is performed by a softmax layer. **Results**: Thanks to the attention mechanisms employed in the developed hybrid model, both the model’s representational power and its classification performance have improved. The model developed to automatically detect and classify maxillary sinus membrane thickening from CBCT images achieved a test accuracy rate of 99.43%. **Conclusions**: The developed model was compared with pre-trained models accepted in the literature and demonstrated superior performance. The current results demonstrate that the proposed model exhibits high performance in classifying maxillary sinus membrane thickness in a single-center CBCT dataset. However, independent external validation and prospective clinical evaluation are necessary to determine its potential for clinical use.

## 1. Introduction

Dental implants are commonly used materials for restoring function and aesthetics in edentulous areas. Insufficient bone volume is a common problem in the posterior maxilla following tooth extraction due to alveolar bone resorption and sinus pneumatization [1]. However, sufficient bone volume is essential for dental implant stability [2]. Sinus lift procedures, which involve elevating the sinus membrane using a crestal or lateral window approach to achieve sufficient bone volume, are commonly performed in dental implantology [3,4]. Complications such as membrane thickening from chronic or acute causes, membrane perforation, and sinusitis resulting from ostial obstruction affect the success of sinus lift procedures [5,6].

The sinus membrane in a healthy sinus is, on average, 0.8 to 1 mm thick and does not present radiological findings [7]. Reports in the literature indicate that membrane thickness can increase by 10–15 times. Many factors trigger membrane thickening, including odontogenic infections, periodontal diseases, endodontic lesions, cystic formations, allergic rhinitis, viral or bacterial infections, smoking, and anatomical variations [8]. In their study, Janner et al. considered membrane thickness above 2 mm to be pathological, classifying it as mild (2–5 mm), moderate (5–10 mm), and severe (over 10 mm) mucosal thickening [9]. Lin et al. reported that a membrane thickness greater than 5 mm caused ostial obstruction in 59.3% of patients, increasing the risk of sinusitis in implant surgery [5]. Studies have also reported that thin or thickened sinus membranes increase the risk of membrane perforation during sinus lift operations, jeopardizing surgical success [10,11]. In addition to the thickness of the sinus lining, other aspects such as its elasticity or fibrosis also affect the risk of perforation.

For sinus lift procedures to be successful and complications to be avoided, the maxillary sinus must be evaluated using CBCT before implant surgery. CBCT is an important diagnostic technique for determining sinus membrane thickness, ostium patency, and detecting sinus diseases since it produces three-dimensional high-resolution pictures at lower dosages [12]. The maximum point of maxillary sinus mucosal thickness may be precisely measured thanks to CBCT’s three-dimensional capabilities, which are essential for identifying minute changes that might not be apparent in two-dimensional pictures [13,14]. In order to visualize the anatomical structure, changes, and diseases of the maxillary sinus, CBCT examination has become a standard procedure in dental implant surgery [15]. However, in clinical practice, performing these measurements manually in each section is time-consuming and may vary depending on the surgeon’s experience [16].

The goal of the diverse field of artificial intelligence (AI) is to carry out a variety of specialized tasks that call for human intelligence. Through the use of deep learning approaches, AI algorithms have improved these learned features over time, mimicking human intelligence [17]. As a subset of deep learning (DL) techniques, convolutional neural networks (CNNs) are capable of automatically identifying fundamental features and classifying them according to the collected data [18]. Convolutional neural networks (CNNs) and Vision Transformer (ViT) architectures, in particular, have provided high accuracy rates in the automatic segmentation and classification of complex anatomical structures. The application of AI and deep learning algorithms in the field of medical imaging has rapidly expanded in recent years.

The aim of this study is to propose a hybrid artificial intelligence model that automatically detects and classifies maxillary sinus membrane thickening in CBCT images. A hybrid model has been developed to automatically detect maxillary sinus membrane thickening from CBCT images. The proposed model enriches the features extracted from a CNN backbone by applying preprocessing to the input images, in parallel with SE, CBAM, and ECA attention mechanisms. These attentive feature representations are combined (concatenated) after Global Average Pooling to obtain a more distinctive feature vector. Then, classification is performed on the regularized MLP header using Dropout. The developed model aims to strengthen inter-class differentiation and improve generalization by integrating the complementary advantages of multiple attention mechanisms within a single framework.

## 2. Materials and Methods

### 2.1. Dataset

**Figure 1:**
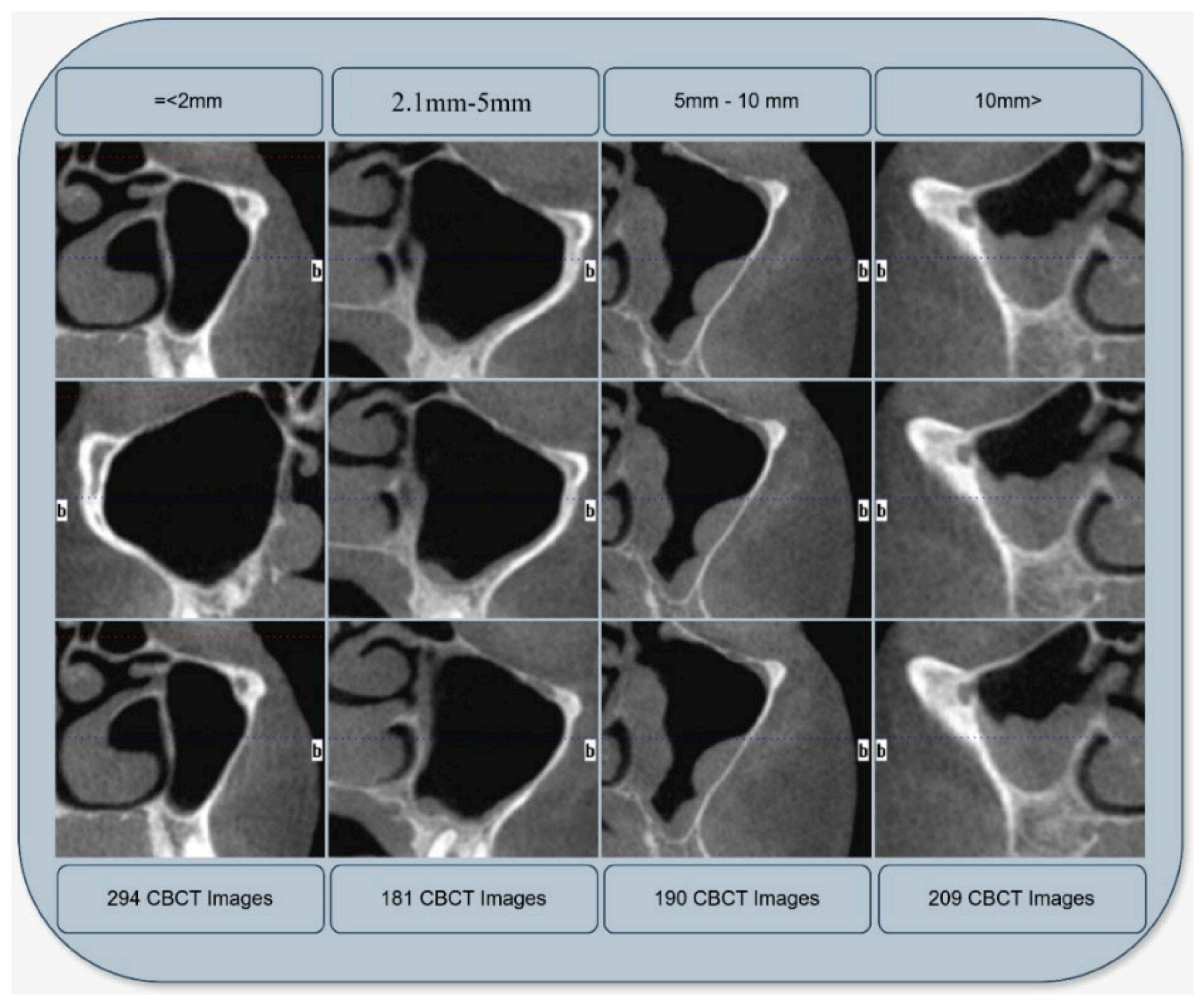
Sample images and numbers for each class.

This retrospective study was approved by the Firat University Non-Interventional Ethics Committee (FÜGOEK) (No. 2025-17-36, Date: 27 November 2025) and conducted in accordance with the Helsinki Declaration. Thirty thousand CBCT images of thirty thousand patients who applied to the Firat University Faculty of Dentistry between January 2019 and September 2025 were examined. These images were obtained using a ProMax 3D Mid (Planmeca, Helsinki, Finland) model tomography device with an exposure time of 8–9 s at 90 kVp and 8 mA. The voxel size of the images was 0.4 mm. Images were analyzed by a specialist physician using Romexis Viewer 4.4.3 (Planmeca, Helsinki, Finland) software with a multiplane reconstruction window that allows viewing the axial, coronal, and sagittal planes at 0.2 mm intervals, according to the manufacturer’s recommendations. The examined images were classified as healthy, 2–5 mm, 5–10 mm, and >10 mm, creating a dataset of 874 images in total. FÜGOEK eliminated the need for informed patient consent. Data were anonymized to prevent patient identification. The dataset contains 294 <2 mm CBCT images, 181 2.1 mm–5 mm CBCT images, 190 5 mm–10 mm CBCT images, and 209 >10 mm CBCT images. The dataset was divided into training and validation sets at the patient level. Images from the same patient were not allowed to be in different data sections. All images from a patient were kept only within the same fold. This prevented related images from the same patient from being present in both the training and validation data, thus preventing patient-related data leakage. Sample images and numerical distributions for each class are shown in Figure 1.

### 2.2. Proposed Model

This study utilized a dataset containing gray-level CBCT images from four classes. The images were first rescaled to standardize their spatial dimensions and converted from a single-channel format to a three-channel format to meet pre-trained CNN input requirements. The CNN models used in this study were not trained from scratch. These models were initialized with pre-trained weights on the ImageNet dataset. Data enhancement techniques, such as random horizontal flips and small angular rotations, were applied during training to improve the model’s generalization. The dataset was divided into training and validation subsets.

#### 2.2.1. Baseline CNN Models

Prior to the proposed approach, current CNN models with different architectural features were used as a basis for comparative evaluation. In this context, DenseNet121 [19], EfficientNetB0 [20], MobileNetV3Large [21], and ResNet50 [22] architectures were considered. All models were initialized with weights pre-trained on ImageNet, and the classification layers were rearranged to suit the problem size. Thus, the performance of architectures with different depths, parameter counts, and connection strategies was evaluated in a consistent training environment.

#### 2.2.2. Proposed Parallel Attention-Based Architecture

**Figure 2:**
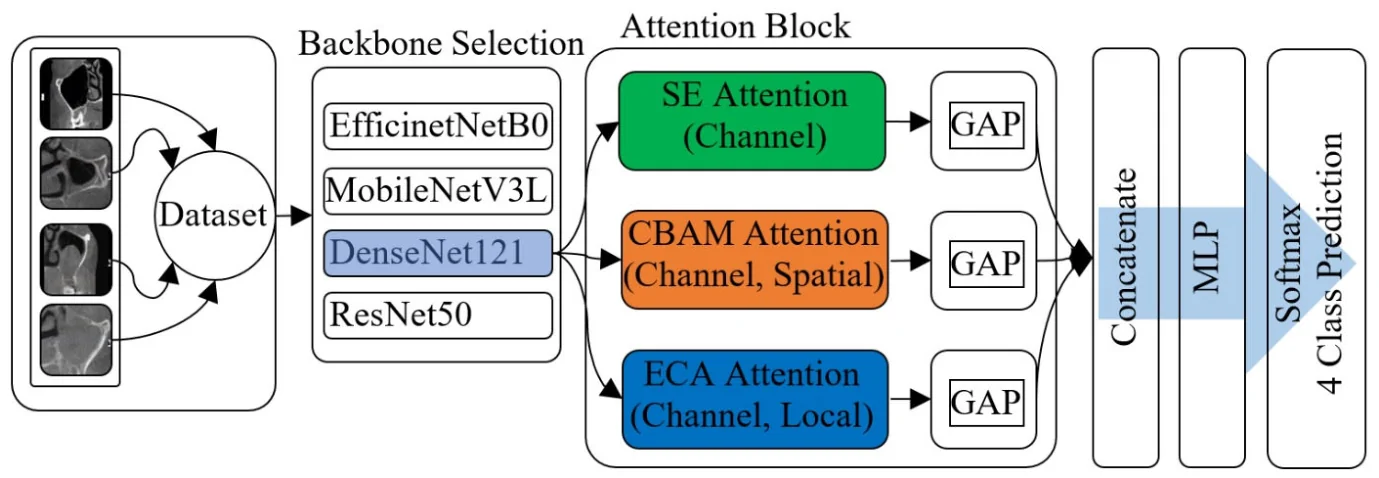
Flowchart of the proposed method.

The DenseNet121 architecture, selected from among the baseline models, is used as the backbone in the proposed model. The final feature map of DenseNet121 is fed into a structure where three different attention mechanisms are applied in parallel. These attention mechanisms consist of Squeeze-and-Excitation (SE) [23], which models channel attention; Convolutional Block Attention Module (CBAM) [24], which considers channel and spatial attention components together; and Efficient Channel Attention (ECA) [25], which emphasizes local channel relationships. Each attention module operates independently on the same feature map and produces its own attention-enhanced feature representations. These representations are converted into vector form using a Global Mean Pooling (GAP) operation and then combined to obtain a single feature vector. This combined vector is passed through a dropout layer to prevent overfitting and transferred to the Multilayer Perceptron (MLP)-based classification header. In the final stage, multi-class prediction is performed using the softmax function. The general flowchart of the proposed architecture is presented in Figure 2.

All models were trained in the Google Colab environment using PyTorch 2.8.0 and timm 1.0.22 libraries. The AdamW algorithm was used for optimization, and the cross-entropy loss function was employed for the multi-class classification problem. Throughout the training process, the behavior of the proposed hybrid model was monitored on both training and validation data. Performance evaluations were also conducted based on standard classification metrics. To assess the consistency of our proposed DenseNet121-based parallel attention-based model against different data splits, the stratified K-fold cross-validation technique was used in the study. The K-fold cross-validation technique ensures the preservation of class distributions in each fold, contributing to more reliable and generalizable results. The model was trained independently in each fold, and performance measurements were recorded. Results for each fold were reported separately, and average performance values were used in comparative analyses.

## 3. Experimental Result

This section presents the results of the proposed parallel attention-based architecture and the results of the models used to compare the performance of the proposed model. Experiments were carried out on an NVIDIA T4 GPU in the Google Colab environment using the Python 3.12.12 programming language. All images were processed at 224 × 224 pixels, and the batch size was determined as 32. The models were trained for a maximum of 20 epochs using early stopping. For optimization, the AdamW algorithm with an initial learning rate of 3 × 10^−4^ and a weight decay value of 1 × 10^−4^ was used, and cross-entropy loss was preferred as the loss function. The training and evaluation processes of the models were carried out using PyTorch and timm libraries. The dataset was divided into training and validation sections, and model performances were evaluated using different metrics. Experimental results were evaluated considering accuracy, weighted precision, recall, and F1-score metrics, as well as confusion matrices and computation times for each model. Examination of the obtained performance measurement metrics showed that the proposed model was more successful. In addition, cross-validation results revealed that the model produced consistent and stable results against different data splits. The images were initially in a single-channel format, and each was converted to three channels to ensure compatibility with pre-trained CNN models. Data enhancement techniques, such as random horizontal flipping and ±10° angular rotation, were applied during training to increase data diversity. These preprocessing steps contributed to the model’s more effective learning of inter-class distinguishing features. Performance measurement metrics are presented in Equations (1)–(4).

Accuracy: (TP + TN)/(TP + TN + FP + FN)(1)

Precision: TP/(TP + FP)(2)

Recall: TP/(TP + FN)(3)

F1-Score: 2∙(Precission∙Recall)/(Precission + Recall)(4)

**Figure 3:**
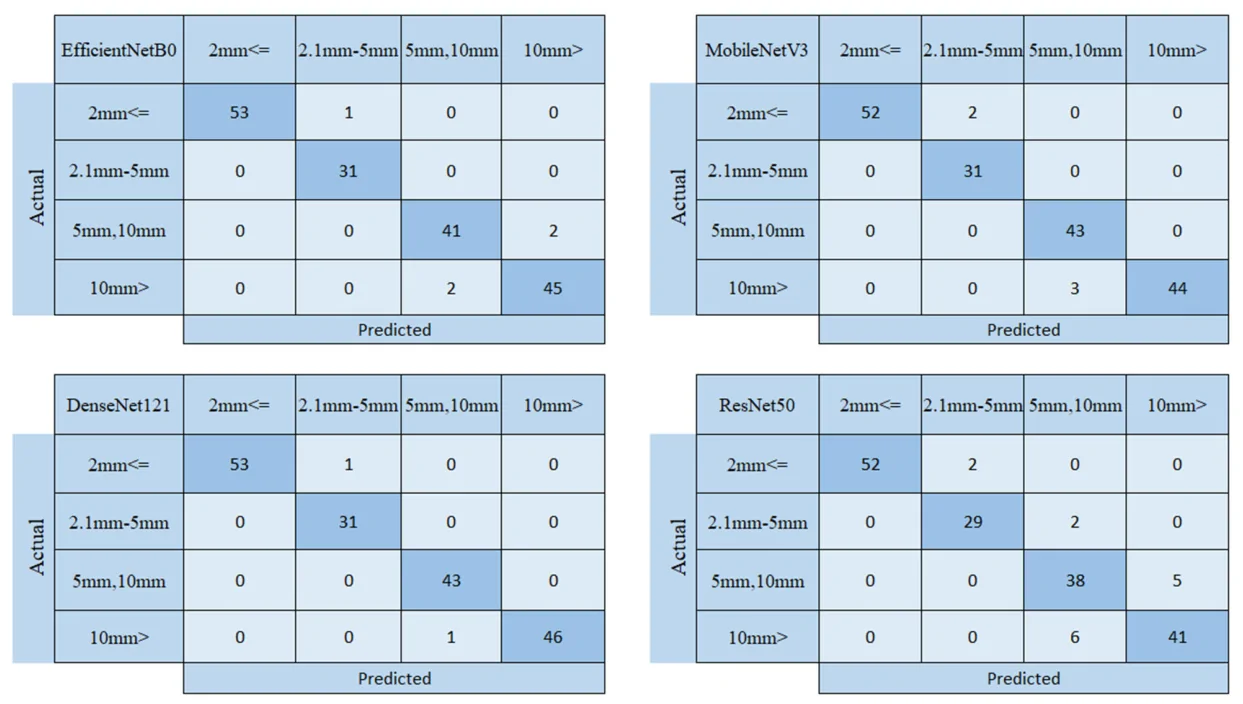
Confusion matrices of pre-trained models.

Results were also obtained using pre-trained models to compare the proposed model’s performance. The confusion matrices obtained from the pre-trained models are presented in Figure 3.

### 3.1. Result of Pre-Trained Model

Examining the confusion matrix for the EfficientNetB0 model in Figure 3, we see that the model’s overall success is high. It was found to have negligible errors between the 10 mm> and 5 mm–10 mm classes. As seen in the matrix, 2 samples belonging to the 5 mm–10 mm class were incorrectly predicted as the 10 mm> class. There are also shifts from the 10 mm> class to other classes. The model gave quite stable results, with 0 errors in the 2.1 mm–5 mm class and 1 error in the 2 mm< class. In addition to these successful results, it was observed that the discriminative power decreased at boundary values where the dimensions were close to each other. When the results of the MobileNetV3-Large model are compared with those of EfficientNetB0, it is similar. The model correctly separated the classes to a large extent, but it made 3 erroneous predictions when classifying samples belonging to the 10 mm> class. Based on the observations, these samples were misclassified as the 5 mm–10 mm class. Additionally, there are 2 erroneous classifications in the <2 mm class. Overall, the MobileNet architecture shows reduced success when handling fine details in X-ray images and when class differences are small.

When the confusion matrix of DenseNet121 is examined, it is seen that the proposed model is more successful than the models used for comparison. DenseNet121 classified test images in the 2.1 mm–5 mm and 5 mm–10 mm classes without error. Only 2 erroneous predictions were observed in total, 1 in the >10 mm class and 1 in the <2 mm class. This result shows that the DenseNet121 architecture is more successful than other CNN models in learning the details in the dataset thanks to its in-depth feature reuse. When the confusion matrix of the ResNet50 model is examined, it is seen that ResNet50 has the highest error rate in the study. When the confusion matrix of the ResNet50 model is examined, a significant discrimination problem was experienced between the “>10 mm” class and the 5 mm–10 mm class. 6 test images in the >10 mm class and 5 test images in the 5 mm–10 mm class were erroneously predicted. The results show that the ResNet50 architecture has weak generalization capabilities on this dataset and is the model most affected by inter-class similarities.

### 3.2. Proposed Hybrid-Based Parallel Attention Model Results

**Figure 4:**
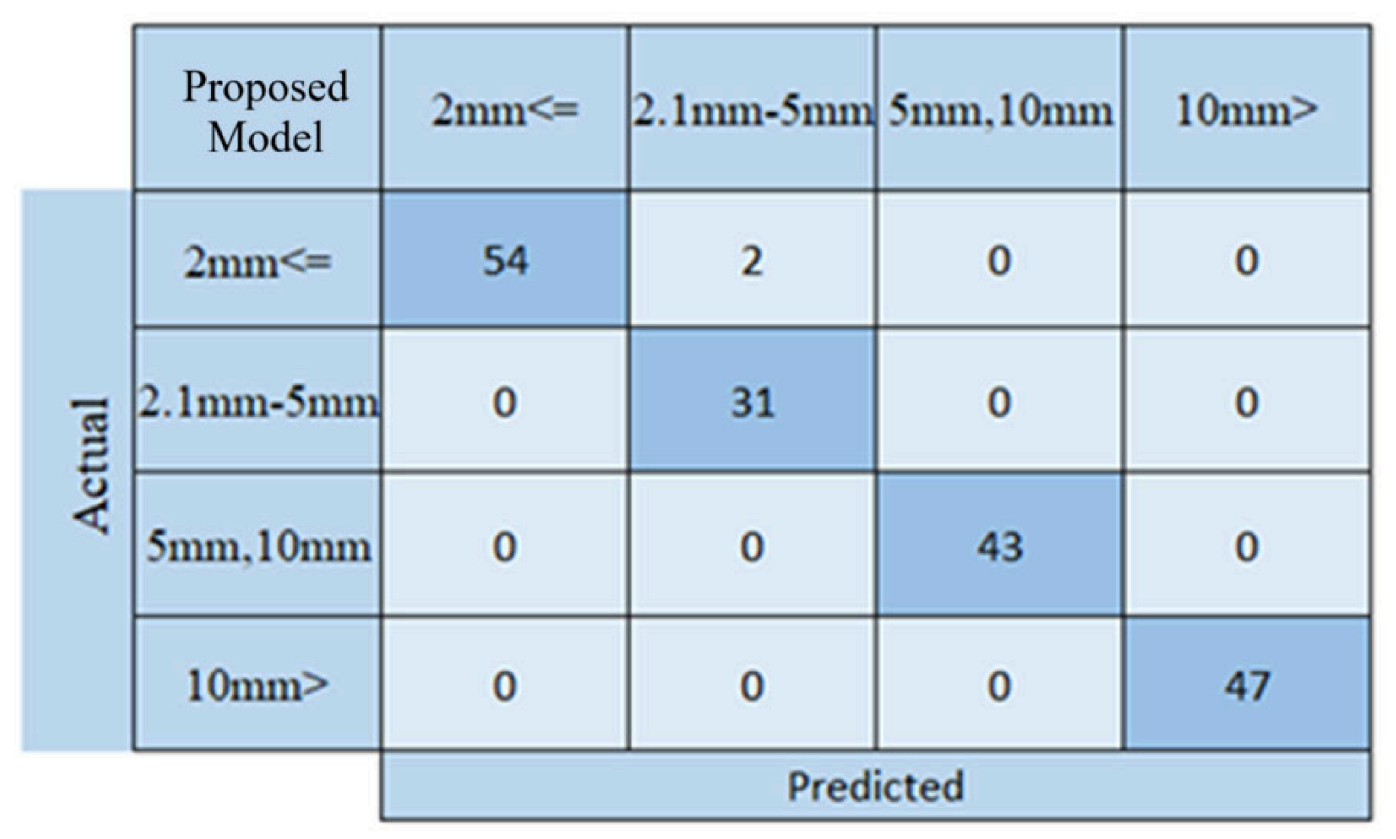
Proposed model best-fold confusion matrix.

The confusion matrix for the best-fold result of the proposed parallel attention-based architecture is shown in Figure 4. Examination of the matrix reveals that predictions for all classes are made with 0 errors. The diagonal values (47, 31, 54, 43) are equal to the total number of samples in each class, indicating that no erroneous predictions were made. This shows that the SE, CBAM, and ECA blocks, which were later added to the model, prevented the errors made by the DenseNet121 model, thus improving its success. Thanks to this hybrid development, the model correctly classified all samples without errors, and its efficiency increased.

Table 1 presents the performance measurement metrics of other models used in the study to allow comparison with the results of the proposed model. Additionally, the 5-fold cross-validation results of the proposed model are also presented in Table 1. Examination of Table 1 shows that the model achieved accuracy rates ranging from 98.86% to 100.00% across folds. In Fold4 iteration, the proposed model achieved 100.00% success. This indicates that the model learned the general characteristics of the dataset rather than memorizing a specific subset. Examining Fold1–Fold5 in terms of “Avg epoch time,” the values in the column ranged from 3.66 s to 3.72 s. These results show that the training process progressed consistently and was balanced in terms of computational load.

Performance metrics and inference times for all models used in the study are presented in Table 2.

Looking at the Accuracy column, a hierarchical performance ranking is observed: ResNet50 (91.43%) < MobileNet/EfficientNet (97.14%) < DenseNet121 (98.86%) < Proposed Model (99.43%). The proposed model also increased the performance of the DenseNet121 architecture, on which it is based, by approximately 0.6% in terms of F1-Score. Examining the last column of the table, “Inference per image,” it is seen that the proposed model has a processing time of 4.00 ms per image. This is a negligible increase of 0.22 ms compared to the base model’s time of 3.78 ms. Considering the accuracy gain obtained, it shows that the proposed method is also an efficient solution for real-time applications. The comparative performance and statistical evaluation of the proposed model and DenseNet121 are presented in Table 3.

According to the five-fold cross-validation results, the proposed model achieved a 0.69% increase in Accuracy and Weighted F1 values and a 0.74% increase in Macro F1 values compared to DenseNet121. Furthermore, lower standard deviation values indicate that the proposed model produces more stable results across folds. The inference times of the two models were also found to be quite similar. These results demonstrate that the proposed structure improves classification performance and stability without significantly increasing computational cost.

## 4. Discussion

In this study, the detection and classification of sinus membrane thickening in CBCT images of the maxillary sinus were evaluated using different AI-assisted models. Among the architectures used for comparison, the DenseNet121 model achieved the highest accuracy of 98.86%. However, with our proposed parallel attention-based model, an accuracy of 99.43% was achieved in classifying sinus membrane thickness. The high performance achieved in the proposed model may stem from the complementary features of the parallel attention mechanisms of DenseNet121. Thanks to its dense connectivity, the DenseNet121 architecture allows for the reuse of learned features at different levels, contributing to the preservation of subtle structural differences between membrane thickness classes. Additionally, SE emphasizes informative channel features, while CBAM evaluates channel and spatial information together. ECA strengthens local inter-channel relationships with low computational cost. The parallel use of these three attention mechanisms, instead of relying on a single attention approach, allows for the joint evaluation of complementary feature representations, contributing to more effective separation of closely related membrane thickness classes.

Evaluating sinus pathologies in dental implant surgeries can be challenging and time-consuming for inexperienced physicians. Despite potential drawbacks such as positioning errors and superimposed images when detecting mucosal thickening in panoramic radiographs, CNN-based deep learning has been reported to achieve promising diagnostic accuracy [26]. For sinus lifting operations performed before implant surgery in the posterior region associated with the maxillary sinus, CBCT is still considered the gold standard compared to traditional two-dimensional radiography techniques to reduce the risk of intraoperative sinus membrane perforation and postoperative sinusitis [14,15,27]. Consistent with the literature, high accuracy rates were obtained using CBCT images for sinus membrane thickness classification.

In the literature, studies related to the maxillary sinus have been conducted using different deep learning models for diagnosis, segmentation, and classification from panoramic, Waters radiographs, CBCT, and CT sections [27,28,29,30]. Jung et al., in their segmentation study of maxillary sinus lesions using a CNN-based 3D nnU-Net deep learning architecture, found DSCs values of 0.92 ± 0.17. They also stated that AI-assisted segmentation was completed more quickly than manual segmentation [31]. Ha et al., in a retrospective study using a CNN-based EfficientDent architecture, reported a 92% accuracy in diagnosing pseudocysts on panoramic radiographs [26]. Hung et al. In studies that used V-Net and SVR model architectures to detect maxillary sinus membrane thickening and mucosal retention cysts in low- and full-dose CBCT images, researchers reported AUC values of 91% and 93%, respectively [32]. In a similar study, He et al. used a deep CNN model in a segmentation study with a dataset of 175 CBCT images that they had previously classified as healthy, 2 mm–4 mm, 4 mm–10 mm, and >10 mm, and stated that the model showed satisfactory results [33]. In a study in which Serindere et al. diagnosed sinusitis using a CNN-based artificial intelligence model on panoramic radiographs and CBCT images, they found an accuracy of 75.7% for panoramic radiographs and 99.7% for CBCT images [27]. In this study, we detected maxillary sinus membrane thickenings using CBCT sections and an AI-assisted hybrid model (CNN-ViT), classifying them into four different groups (healthy, 2–5 mm, 5–10 mm, and >10 mm) with a 99.43% accuracy rate. In a systematic review, Wu et al. stated that model performance varies with dataset diversity, imaging method, and architecture but generally provides high diagnostic accuracy. Given the limited number of studies on the classification of sinus membrane thickenings, the 99.43% accuracy rate is high compared to other studies in the literature [34].

This study, like all studies, has some limitations. The study was conducted with data obtained from a single center. It is necessary to test the model’s performance with different devices in other centers. In addition, the analyses were performed on cross-sections of three-dimensional volumetric data. Performing three-dimensional volumetric analysis in future studies may further increase the success rate. Additionally, using data from different centers is important for generalizing the model. Nevertheless, the developed artificial intelligence model has the potential to be a powerful tool for dentists in pre-operative planning and reducing the risk of complications by detecting and classifying maxillary sinus membrane thickening with high accuracy.

## 5. Conclusions

This study aims to automatically classify X-ray images. To this end, an innovative deep learning model based on the DenseNet121 architecture, combining SE, CBAM, and ECA blocks in parallel, has been developed. The proposed hybrid method was applied to a 4-class dataset consisting of 874 images. The effectiveness of the proposed hybrid method was validated with the results obtained. The proposed model was found to achieve an accuracy rate of 99.43%. This value demonstrates that it is more successful than accepted models in the literature such as EfficientNetB0, MobileNetV3Large, and ResNet50. The proposed hybrid model reduced the misclassification rate by focusing on key regions in feature maps. In conclusion, the high accuracy rate and low computational load achieved in the developed hybrid model show that the proposed model successfully detects sinus membrane thickness. Our future goals include testing the proposed model on larger and more diverse datasets.

## Figures and Tables

**Table 1:** K-fold validation result.

Fold	Accuracy	Weighted Precision %	Weighted Recall %	Weighted F1 Score %	Avg. Epoch Time (s)	Inference per Image (ms)
Fold 1	98.86	98.92	98.86	98.86	3.66	4.05
Fold 2	99.43	99.44	99.43	99.43	3.68	4.05
Fold 3	99.43	99.44	99.43	99.43	3.62	3.94
Fold 4	100.00	100.00	100.00	100.00	3.64	3.94
Fold 5	99.43	99.44	99.43	99.43	3.72	4.01

**Table 2:** Performance metrics comparison for all models.

Model	Accuracy %	Weighted Precision %	Weighted Recall %	Weighted F1 Score %	Inference per Image (ms)
Proposed Model (DenseNet121 + 3Attn) Cross Validation	99.43	99.45	99.43	99.43	4.00
DenseNet121	98.86	98.89	98.86	98.86	3.78
EfficientNetB0	97.14	97.16	97.14	97.15	3.84
MobileNetv3L	97.14	97.32	97.14	97.15	3.71
ResNet50	91.43	91.67	91.43	91.51	3.51

**Table 3:** Comparative performance and statistical evaluation of the proposed model and DenseNet121.

Metric	Proposed Mean ± SD	95% CI	DenseNet121 Mean ± SD	95% CI	Difference
Accuracy	99.43 ± 0.40	98.93–99.93	98.74 ± 0.85	97.68–99.80	+0.69
Weighted F1	99.43 ± 0.40	98.93–99.93	98.74 ± 0.84	97.70–99.78	+0.69
Macro F1	99.36 ± 0.48	98.76–99.96	98.62 ± 0.92	97.48–99.76	+0.74
Inference time	3.9948 ± 0.0541	3.9276–4.0620	3.9914 ± 0.1720	3.7778–4.2050	+0.0034 ms

## Data Availability

Data used in this research are available upon reasonable request from the corresponding author.
